# The Effects of Creatine Supplementation on Explosive Performance and Optimal Individual Postactivation Potentiation Time

**DOI:** 10.3390/nu8030143

**Published:** 2016-03-04

**Authors:** Chia-Chi Wang, Ming-Ta Yang, Kang-Hao Lu, Kuei-Hui Chan

**Affiliations:** 1 Office of Physical Education, National Taipei University of Business, Taipei 10051, Taiwan; sunnywango@gmail.com; 2 Graduate Institute of Athletics and Coaching Science, National Taiwan Sport University, Taoyuan 33301, Taiwan; yangrugby@gmail.com; 3 Sports Science and Research Department, National Sports Training Center, Kaohsiung 81343, Taiwan; 980506@ntsu.edu.tw

**Keywords:** complex training, counter movement jump, peak power, maximal muscle strength

## Abstract

Creatine plays an important role in muscle energy metabolism. Postactivation potentiation (PAP) is a phenomenon that can acutely increase muscle power, but it is an individualized process that is influenced by muscle fatigue. This study examined the effects of creatine supplementation on explosive performance and the optimal individual PAP time during a set of complex training bouts. Thirty explosive athletes performed tests of back squat for one repetition maximum (1RM) strength and complex training bouts for determining the individual optimal timing of PAP, height and peak power of a counter movement jump before and after the supplementation. Subjects were assigned to a creatine or placebo group and then consumed 20 g of creatine or carboxymethyl cellulose per day for six days. After the supplementation, the 1RM strength in the creatine group significantly increased (*p* < 0.05). The optimal individual PAP time in the creatine group was also significant earlier than the pre-supplementation and post-supplementation of the placebo group (*p* < 0.05). There was no significant difference in jump performance between the groups. This study demonstrates that creatine supplementation improves maximal muscle strength and the optimal individual PAP time of complex training but has no effect on explosive performance.

## 1. Introduction

Numerous studies have investigated combined nutritional supplementation with exercise or training programs for enhancing an athlete’s performance. Creatine (Cr) is one of the most popular and widely researched natural supplements designed to increase exercise-related strength and power [1]. Studies have demonstrated some positive effects of short-term (5–7 days) Cr supplementation on exercise [2,3]. A number of review articles have also reported that Cr supplementation can significantly increase strength, power, and/or work performance during multiple sets of maximal effort muscle contractions [1,4].

Complex training generally involves a heavy resistance-training exercise followed by a biomechanically similar plyometric exercise [5]. Recently, complex training has been suggested to be an effective training method for enhancing power output in athletes [6]. The physiological rationale behind complex training has been termed postactivation potentiation (PAP) [7,8]. PAP refers to the phenomenon by which the acute contractile ability of a muscle is enhanced in response to a conditioning stimulus such as a heavy resistance exercise (HRE). Therefore, the effects of PAP have been used in pre-exercise warm-up, complex training programs because of its positive acute effect on increasing motor performance and its chronic effect on enhancing strength and power [6,9,10,11,12]. However, studies have reported different and contradictory results. Some studies have indicated that explosive performance is enhanced within 3–5 min after HRE [13,14,15], whereas other studies have not shown a positive effect when explosive performance was performed immediately after the HRE [16,17,18]. These conflicting results may be because PAP is influenced by muscle fatigue and is an individualized phenomenon.

Both fatigue and PAP appear to be at their maximum and coexist immediately after the HRE stimulus [9,11,13,19,20,21]. Fatigue is more dominant in the early stage of recovery and masks the effects of PAP. Consequently, subsequent exercise performance is diminished or unchanged [7,8,9]. For example, a shorter recovery time (<15 s) causes a decline in the jump performance after HRE [11,20,22,23]. Two principal mechanisms for the development of fatigue during intensive muscle contraction are the rephosphorylation of adenosine diphosphate (ADP) and an increase in the concentration of hydrogen ions (H^+^) resulting from the dissociation of lactic acid, which lead to a decrease in phosphocreatine (PC) [24]. In this scenario, one could speculate that optimizing energy provision would be an essential factor in mitigating the negative effects of fatigue on PAP. Cr plays an important role in skeletal muscle energy metabolism. Cr supplementation could aid the rephosphorylation of ADP to adenosine triphosphate (ATP) and improve cellular homeostasis during bouts of intense activity by utilizing the H^+^ produced during the process of creatine kinase reaction to buffer the pH [1]. A previous meta-analysis reported that Cr supplementation had a pronounced effect on short-duration (<30 s) high intensity exercises [25]. In this regard, Cr supplementation may have positive effects on reducing muscle fatigue after HRE. Additionally, Cr supplementation may facilitate the reuptake of calcium ions into the sarcoplasmic reticulum via calcium pumps [26]. This may generate force more rapidly during short-duration, predominantly anaerobic, intermittent exercises due to faster detachment of the actomyosin bridges [4].

It seems that optimal performance occurs when fatigue has subsided, while the potentiated effect still exists [6]. Thus, the precise recovery time is essential. Recent studies have focused on optimal PAP recovery time for warm-ups between HRE and explosive performance and have attempted to identify the precise recovery time needed to elicit a PAP effect during warm-up periods [11,13,19,20,27]. However, there is no consistent agreement on the required recovery time. Studies have suggested that recovery durations of 5 min [13], 8 min [19], 4–12 min [20], 7–10 min [27] and 8–16 min [11] may elicit PAP, leading the authors to conclude that PAP is a highly individualized phenomenon [11,13,19,20,28,29]. Individual backgrounds (training experience, level of performance, and predominance of fast twitch fibers *etc.*) can also affect the PAP response. Therefore, the application of individual recovery times (optimal individual PAP times) is an effective approach. Nonetheless, very limited studies have investigated the issue of optimal recovery time between HRE and explosive performance.

To our knowledge, no study has investigated the effects of Cr supplementation on increasing PAP effects during a complex training bout. This study aimed to examine the effects of short-term Cr supplementation on strength and power performance as well as the optimal individual PAP time during a complex training bout. We hypothesized that Cr supplementation would increase maximal muscle strength and power performance, as well as shorten optimal individual PAP time during a complex training bout.

## 2. Experimental Section

### 2.1. Research Design

Before formal measurements, all subjects visited the laboratory initially to ensure familiarity with the back squat and the countermovement jump (CMJ) technique. Subjects were educated on the familiarization session by a well-trained fitness instructor. They conducted 2–10 times of CMJ until correct technique can be performed. Thereafter, subjects completed 2–5 sets of 6–10 repetitions using only a men’s Olympic bar (20 kg) to familiarize with the squat movement technique, 1 min interval for recovery was used in each set.

The day after familiarization session, the anthropometry and the strength of one repetition maximum (1RM) back squat were measured. Two days later, subjects also performed two sets of complex training bouts to determine the individual optimal timing of PAP, height and peak power (PP) of CMJ in another two days. A double-blind, randomized matched-pair design was used to assign 30 subjects from three explosive type sports to control for two potential variables (different training routines of teams and 1RM strength) into a Cr or placebo (Pla) group. After six days of high dose Cr or Pla supplementation, the same test procedures before supplementation were conducted again to evaluate the effects of Cr supplementation. Meanwhile, a low dose of Cr or Pla supplementation was maintained until the end of the study. All familiarization and experimental sessions of this study were performed at the same time (from 10:00 to 14:00) of each day.

### 2.2. Subjects

Thirty male university athletes from baseball, basketball, and tchoukball teams (10 for each sport) volunteered to participate in this study. The characteristics of the subjects are described in Table 1. The study was approved by the Institutional Review Board of the Taoyuan General Hospital, Taiwan. All subjects provided written informed consent before participation. They maintained their basic training programs and were asked to keep their normal diet pattern during the experimental period. Subjects were excluded if they had one of the following: (1) a maximal squat strength less than 1.5 times their body weight [30]; (2) injury to a lower limb within the past six months; (3) the experience of both back squat and plyometric training within less than 1 year; or (4) have taken chronic or daily doses of anti-inflammatory medications or nutritional supplements within the past month.

### 2.3. Supplementation Protocol

After the baseline testing, subjects in the Cr group (Cr gr.) began consuming 5 g of creatine monohydrate (creatine fuel powder; Twinlab, CO, USA) plus 5 g of dextrose dissolved in 300 mL of water four times (at breakfast, lunch, dinner and before bedtime) per day for six days. Subjects in the Pla group (Pla. gr.) followed the same protocol but consumed carboxymethyl cellulose (food grade CMC powder, GreenYoung Co., Taichung, Taiwan) instead of Cr. The supplements for both groups were the same color and taste. For maintenance, subjects ingested single daily doses of 2 g of creatine monohydrate or carboxymethyl cellulose powder plus 2 g dextrose after lunch until the end of the study.

### 2.4. Prediction of One Repetition Maximum Strength

Prediction of 1RM strength for the back squat was determined based on the protocol described by Baechle *et al.* [31]. In brief, subjects jogged for 5 min on a treadmill followed by lower/upper limb light stretching exercises and two light resistance warm-up sets. After 1 min of rest, the subjects executed a load of 87%–93% of the predicted 1RM through the full range of motion. After each successful performance, the load was increased in increments of 14–18 kg until only one successful repetition could be completed. Four min rests were given between each test. The increase or decrease in the load continued until the subject was able to complete one repetition with the proper exercise technique. Ideally, the subject’s 1RM was measured within five testing sets.

### 2.5. Optimal Individual PAP Time and Jump Performance Test

After a low intensity aerobic exercise at a comfortable pace followed by a lower limb light stretching exercise for warm-up, subjects performed two CMJs with a 5 s rest interval for baseline. During the CMJ test, subjects jumped on a force plate (Smartspeed™, Fusion Sport, Brisbane, Australia) with hands placed on their hips at all times. Each jump was separated by a 5 s rest period. The best performance of jump height and its peak power (PP) output was used in the analysis.

After a 5 min rest, subjects executed a set of complex training bouts with 5RM back squat exercises to elicit PAP [32] followed by a counterbalanced order of the six rest intervals (1, 3, 5, 7, 9, 11 min or 2, 4, 6, 8, 10, 12 min) for two days. The optimal individual PAP time was the rest interval with the maximum delta-values of *jump height* for complex training bouts minus baseline values.

### 2.6. Anthropometric Measurements

All subjects visited the laboratory in the morning for anthropometric measurements including body height (cm), body mass (kg) and body fat percentage (%). Standing body height without shoes or socks was measured to the nearest 0.1 cm with a height meter mounted on a wall. Body mass and body fat percentage were measured by a bioelectrical impedance instrument (InBody 3.0, Biospace, Seoul, Korea) with standard methods to assess body composition.

### 2.7. Statistical Analysis

Statistical analyses were performed using SPSS version 19.0 software (SPSS Inc., Chicago, IL, USA). Data are expressed as the mean ± SD. An independent-sample *t*-test was used to compare the subjects’ characteristics between the groups. A mixed design two-way ANOVA (group × time) was used to compare the variables of CMJ, PP, the optimal individual PAP time and 1RM strength. The statistical power of the test calculation was obtained by using the equations of Cohen [33]. Statistical significance was set at *p* < 0.05.

## 3. Results

### 3.1. Subject Characteristics

Subject characteristics for both groups are presented in Table 1. No significant differences were noted for any variable (*p* > 0.05).

### 3.2. Effects of Cr Supplementation on Maximum Muscle Strength and Variables in a Set of Complex Training Bout

**Figure 1:**
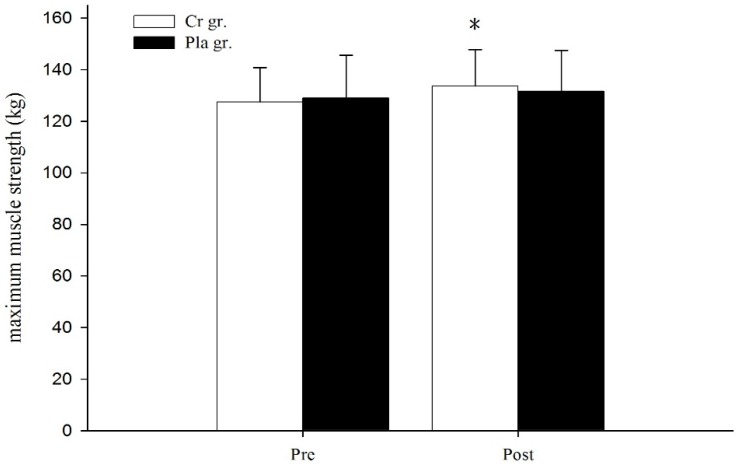
Maximum muscle strength of back squat following creatine or placebo supplementation. Data are the mean ± SD. Asterisk (*) indicates a significant difference (*p* < 0.05) from the pre value within the group.

**Figure 2:**
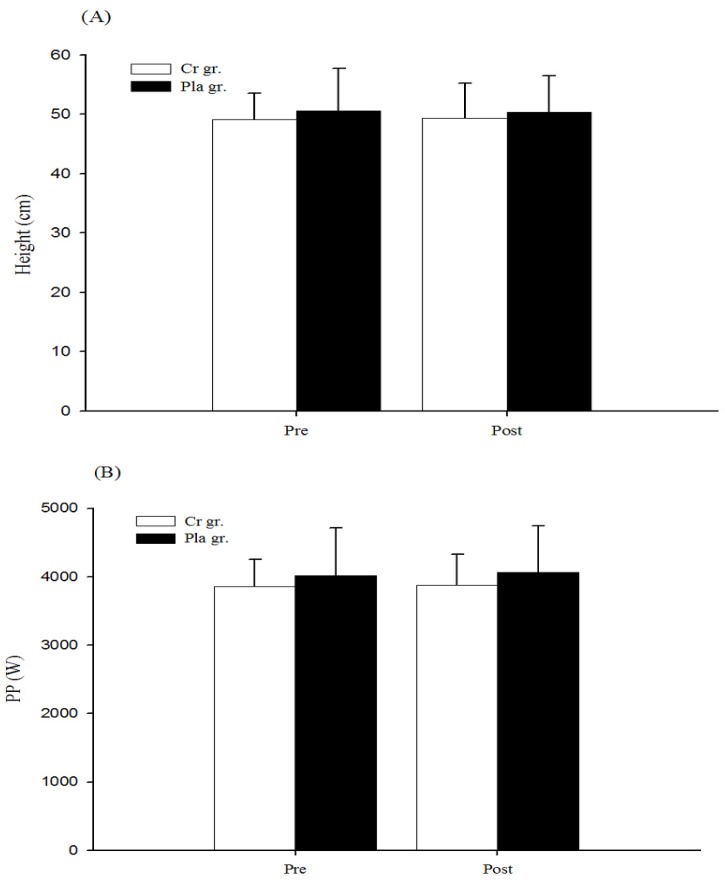
(**A**) Height and (**B**) peak power (PP) of counter movement jump of optimal individual postactivation potentiation (PAP) time following creatine or placebo supplementation. Data are the mean ± SD.

Figure 1 shows the results of 1RM strength before and after six days of Cr or Pla supplementation. Following supplementation, 1RM strength in the Cr gr. significantly increased from 127.33 ± 13.47 kg to 133.67 ± 14.07 kg (*p* < 0.05, statistical power = 1.00). However, there were no significant differences in the Pla gr. or between the Cr and Pla gr. (*p* > 0.05). There was no significant change in height and PP of CMJ from a set of complex training bouts for both groups (*p* > 0.05, Figure 2).

### 3.3. Optimal Individual PAP Time

Table 2 summarizes the time point of the optimal individual time for each individual. The tables illustrate the individual variations in the results. The subjects had their optimal PAP time at different time points in the two groups. Further, the optimal individual PAP time in the Cr gr. was significantly earlier than the pre-supplementation (*p* < 0.05, statistical power = 0.91) and post-supplementation PAP time in the placebo gr. (*p* < 0.05, statistical power = 0.76).

## 4. Discussion

This is the first study investigating the effects of short-term Cr supplementation on sport performances during a set of complex training bouts. Overall, the major findings of this study are that Cr supplementation significantly increased the maximal strength of the lower limbs and reduced the negative influence of fatigue on PAP during a set of complex training bouts. However, this acute benefit could not enhance explosive performance.

Our results show that the optimal individual PAP time was significantly earlier after Cr supplementation. Studies of Izquierdo *et al.* [2] and Urbanski *et al.* [34] reported that five days of Cr supplementation (20 g/day) significantly improved the total repetitions performed to fatigue during repetitive high-power-output exercise bouts and increased the time to fatigue during three bouts of submaximal knee extension. Rahimi *et al.* [3] also found that seven days of Cr supplementation (20 g/day) increased the number of repetitions and the training volume on the 5th of a 6-set of squat exercises. The authors concluded that the mechanisms for these improvements may be due to an increase in the availability of PC to synthesize ATP during contraction, PC resynthesis during recovery and muscle buffering capacity. Taken together, these physiologic changes reduce fatigue and increase the recovery rate from exercise. The outcome of this study was consistent with previous studies [2,3,34,35].

However, our study did not show any significant improvement in explosive performance after Cr supplementation. This result may be explained by certain observations that have been reported in several studies [10,36]. Hamada *et al.* [10] indicated that PAP was induced in the knee extensor muscles by a 10-s maximum voluntary isometric contraction (MVC) and immediately after the MVC. The PAP of twitch peak torque increased with a mean of 70.6% immediately but then rapidly declined to +44% and +31% at 30 s and 60 s, respectively. Potentiation continued to decrease only 12% above the pre-MVC value after 5 min. Similar findings have been reported in another study [36] in which twitch potentiation in the knee extensor muscles after a 7-s MVC was immediately increased and then sharply declined during the first and third minutes of recovery. These findings demonstrated that peak PAP was immediately increased by conditioning contraction, but instantly began to decrease with a rapid decline for 3 min followed by a more gradual decline over the remainder of the recovery period. Therefore, although optimal individual PAP time in our study was earlier from 6.13 min to 4 min, the elicited PAP was not sufficient to enhance the performance of CMJ. Additionally, we found that the 1RM strength back squat increased after Cr supplementation. This result is in agreement with previous studies [2,3,34,37] and reviews [1,38,39]. These studies have examined the acute effects of Cr loading (20 g/day for 5–7 days) on strength performance and generally concluded that the mechanisms underlying this improvement were due to an enhanced capacity of high-energy phosphate diffusion between the mitochondria and myosin heads, thus enabling the heads to engage in cross-bridge cycling and tension maintenance. Other reasons may involve a substantial increase in the rate of ATP synthesis and the amount of power generated during the short duration (in seconds) of a very power-demanding exercise such as 1RM.

Our finding supports the hypothesis that PAP is a highly individualized phenomenon. We found that some subjects had the greatest PAP effect 3–6 min after HRE, whereas this time interval varied for other subjects. Our results are in agreement with a previous study by McCann *et al.* [18] who found that a majority of subjects had their largest PAP in a vertical jump height with 4 min of rest, while a quarter of them (25%) responded better to a 5 min rest interval. Our finding is also in line with Naclerio *et al.* [40] in which the optimal individual potentiation of PAP was displayed over a broad window of potentiation from 1 to 12 min after different volumes of conditioning activity. Comyns *et al.* [30] also reported that subjects had their greatest improvement in flight time and ground reaction force performance at four different rest intervals (30 s, 2, 4 and 6 min). As other studies have suggested, the discrepancy in optimal individual recovery time can be attributed to the differences in the subjects' backgrounds.

## 5. Conclusions

This study provided potentially important information about the effects of short-term Cr supplementation on enhancing maximal muscle strength of the lower limbs and improving the influence of fatigue on the PAP effect during a set of complex training bouts. Moreover, an appropriate individual rest interval may be paramount to optimizing the effectiveness of complex training.

Further studies should evaluate the efficiency of Cr supplementation on other main biomarkers and athletic performances after long-term complex training with optimal individual PAP time and should attempt to combine other nutritional supplements for added beneficial effects.

## Figures and Tables

**Table 1:** Subject characteristics.

Variable	Cr gr. (*n* = 15)	Pla gr. (*n* = 15)
Age (years)	19.93 ± 1.86	19.46 ± 1.12
Height (cm)	171.93 ± 4.86	175.93 ± 8.49
Weight (kg)	66.98 ± 6.74	70.24 ± 11.14
Body fat (%)	15.13 ± 5.06	13.66 ± 4.37

Data are mean ± SD.

**Table 2:** Optimal individual postactivation potentiation (PAP) time for each subjects following creatine or placebo supplementation.

Subjects	Cr gr.		Pla gr.	
	Pre (Min)	Post (Min)	Pre (Min)	Post (Min)
1	3	5	5	3
2	7	3	4	4
3	5	5	4	12
4	11	3	7	5
5	4	6	7	7
6	5	2	5	6
7	4	2	8	10
8	6	5	9	5
9	8	1	3	1
10	6	5	3	6
11	6	5	6	7
12	5	3	8	9
13	12	4	7	10
14	6	4	2	7
15	4	7	2	4
Mean ± SD	6.13 ± 2.53	4.00 ± 1.64 *	5.33 ± 2.28	6.40 ± 2.94 ^#^

Cr gr. = creatine group; Pla gr. = placebo group; Pre = pre-supplementation; Post = post-supplementation. * indicates a significant difference (*p* < 0.05) from the pre value within the group; ^#^ indicates a significant difference (*p* < 0.05) from the post-supplementation of the Cr gr.
